# Supplementary material for: Relations between Sensory Responsiveness and Features of Autism in Children
Source: Brain Sci. 2020 Oct 24;10(11):775. doi: 10.3390/brainsci10110775 (PMC7690864; doi:10.3390/brainsci10110775)
Supplement: Supplementary file 1 [file brainsci-10-00775-s001.pdf]

File S1. Information on flexible use of terminology.

There is some debate in the literature regarding whether researchers should utilize person-first language (e.g., individuals with autism, individuals with ASD) versus identity-first language (e.g., autistic individuals). Clinicians and researchers tend to prefer person-first language, while many autistic individuals and their allies prefer and advocate for identity-first language [1,2]. In this manuscript, we refer to children with autism using person-first language while otherwise avoiding ableist language [3], and acknowledge that this approach may not be received equally by all of the stakeholders of our research.

1. Gernsbacher, M.A. Editorial perspective: The use of person-first language in scholarly writing may accentuate stigma. *J. Child Psychol. Psychiatry* **2017**, *58*, 859–861, doi:10.1111/jcpp.12706.
2. Kenny, L.; Hattersley, C.; Molins, B.; Buckley, C.; Povey, C.; Pellicano, E. Which terms should be used to describe autism? Perspectives from the UK autism community. *Autism* **2016**, *20*, 442–462, doi:10.1177/1362361315588200.
3. Bottema-Beutel, K.; Kapp, S.K.; Lester, J.N.; Sasson, N.J.; Hand, B.N. Avoiding ableist language: Suggestions for autism researchers. *Autism Adulthood* **2020**, doi:10.1089/aut.2020.0014.

Table S1. Partial correlations between patterns of sensory responsiveness and core and related features of autism controlling for nonverbal IQ.

	Hypo	Hyper	Seeking
Core Features of Autism			
SRS Social Communication T-score	.85***	.81***	.57***
SRS RRB T score	.77***	.78***	.69***
Core Autism Feature Aggregate	.87***	.82***	.63***
Related Features of Autism			
VABS Communication SS	–.70***	–.62***	–.51***
VABS Daily Living Skills SS	–.64***	–.61***	–.38**
VABS Socialization SS	–.79***	–.73***	–.52***
BASC Anxiety T score	.42***	.61***	.27*
BASC Depression T score	.62***	.70***	.50***
BASC Somatization T score	.33**	.42***	.14
BASC Internalizing Problem Behaviors T score	.55***	.68***	.37**
BASC Functional Impairment Probability T score	.79***	.80***	.61***
Language			
ROWPVT SS	–.24	–.18	–.28**
EOWPVT SS	–.23	–.18	–.19
CELF Receptive Language SS	–.31*	–.26*	–.32**
CELF Expressive Language SS	–.33**	–.26*	–.34**
CELF Core Language SS	–.35**	–.29*	–.38**

Note. Nonverbal IQ = Nonverbal intelligence as measured by the Leiter International Performance Scale–3rd edition [41] or the Test of Nonverbal Intelligence–4th edition [40], Hypo = hyporesponsiveness aggregate score, Hyper = hyperresponsiveness aggregate score, Seeking = sensory seeking aggregate score, SRS = Social Responsiveness Scale [45], Core Autism Feature Aggregate = Aggregate of SRS total T score and Social Communication Questionnaire total score [43], SS = Standard score, VABS = Vineland Adaptive Behavior Scales–2nd edition [46], BASC = Behavior Assessment System for Children–3rd edition [47], ROWPVT = Receptive One Word Picture Vocabulary Test–4th edition [48], EOWPVT = Expressive One Word Picture Vocabulary Test–4th edition [49], CELF = Clinical Evaluation of Language Fundamentals–4th edition [50]

*$p < .05$, **$p < .01$, ***$p < .001$ after applying a Benjamini-Yekutieli correction [53]
